# Transcriptome and metabolome analyses reveal new insights into chlorophyll, photosynthesis, metal ion and phenylpropanoids related pathways during sugarcane ratoon chlorosis

**DOI:** 10.1186/s12870-022-03588-8

**Published:** 2022-04-29

**Authors:** Ting Luo, Zhongfeng Zhou, Yuchi Deng, Yegeng Fan, Lihang Qiu, Rongfa Chen, Haifeng Yan, Huiwen Zhou, Prakash Lakshmanan, Jianming Wu, Qi Chen

**Affiliations:** 1 Sugarcane Research Institute, Guangxi Academy of Agricultural Sciences, Nanning, Guangxi China; 2 Sugarcane Research Center, Chinese Academy of Agricultural Sciences, Nanning, Guangxi China; 3 Key Laboratory of Sugarcane Biotechnology and Genetic Improvement (Guangxi), Ministry of Agriculture, Nanning, Guangxi China; 4 Guangxi Key Laboratory of Sugarcane Genetic Improvement, Nanning, Guangxi China; 5 Interdisciplinary Research Center for Agriculture Green Development in Yangtze River Basin, College of Resources and Environment, Southwest University, Chongqing, 400716 China; 6 Queensland Alliance for Agriculture and Food Innovation, University of Queensland, QLD, St Lucia, 4067 Australia; 7 Nanning New Technology Entrepreneur Center, Nanning, Guangxi China

**Keywords:** Ratoon sugarcane chlorosis, Chlorophyll metabolism, Photosynthesis, Metal ion metabolism, Phenylpropanoids biosynthesis

## Abstract

**Background:**

Ratoon sugarcane is susceptible to chlorosis, characterized by chlorophyll loss, poor growth, and a multitude of nutritional deficiency mainly occurring at young stage. Chlorosis would significantly reduce the cane production. The molecular mechanism underlying this phenomenon remains unknown. We analyzed the transcriptome and metabolome of chlorotic and non-chlorotic sugarcane leaves of the same age from the same field to gain molecular insights into this phenomenon.

**Results:**

The agronomic traits, such as plant height and the number of leaf, stalk node, and tillers declined in chlorotic sugarcane. Chlorotic leaves had substantially lower chlorophyll content than green leaves. A total of 11,776 differentially expressed genes (DEGs) were discovered in transcriptome analysis. In the KEGG enriched chlorophyll metabolism pathway, sixteen DEGs were found, eleven of which were down-regulated. Two photosynthesis pathways were also enriched with 32 genes downregulated and four genes up-regulated. Among the 81 enriched GO biological processes, there were four categories related to metal ion homeostasis and three related to metal ion transport. Approximately 400 metabolites were identified in metabolome analysis. The thirteen differentially expressed metabolites (DEMs) were all found down-regulated. The phenylpropanoid biosynthesis pathway was enriched in DEGs and DEMs, indicating a potentially vital role for phenylpropanoids in chlorosis.

**Conclusions:**

Chlorophyll production, metal ion metabolism, photosynthesis, and some metabolites in the phenylpropanoid biosynthesis pathway were considerably altered in chlorotic ratoon sugarcane leaves. Our finding revealed the relation between chlorosis and these pathways, which will help expand our mechanistic understanding of ratoon sugarcane chlorosis.

**Supplementary Information:**

The online version contains supplementary material available at 10.1186/s12870-022-03588-8.

## Introduction

Sugarcane is a major food and energy crop globally. In China, sugarcane accounts for 90% of sugar production, contributing 6 to 8 billion RMB annually [1]. In Guangxi, the leading sugar-producing province in China, ratoon sugarcane accounted for 60–70% of the total planting area [2]. Ratooning is critical for reducing crop production costs. However, ratoon crops face various problems. Plantlet chlorosis is one of them which is now widespread in Guangxi. Approximately 40% of the sugarcane cultivated area in Guangxi is now affected, causing production loss ranging from 23 to 40% [2, 3].

In plants, leaf chlorophyll loss causes chlorosis, and it could be due to accelerating chlorophyll catabolism or reduced chlorophyll production, or by both. Chlorophyll metabolism involves a series of enzymes, including HemA, HemB, chlH, chlM, por, and NOL [4]. Iron deficiency affects chlorophyll synthesis, causing chlorosis in *Areca catechu* (Arecaceae) [5]. Low expression of chlorophyll metabolism genes was found in light green cucumber [6]. Chlorophyll is the primary pigment that absorbs light energy for photosynthesis reactions [7, 8]. Low chlorophyll content in chlorotic leaves decreased photosynthesis and fresh weight in *Areca catechu* and cucumber [5, 9]. Chlorophyll biosynthesis plays a vital role in maintaining photosynthetic machinery [10, 11]. Iron is an essential micronutrient for chlorophyll synthesis [12], and its deficiency is the most common cause of chlorosis [13]. Previous reports indicate that the chlorosis symptoms observed in ratoon sugarcane in Guangxi were similar to iron (Fe) chlorosis found in India, yet, the causal factors appear different [14–16]. Excessive Manganese (Mn) had been found in ratoon sugarcane chlorotic plantlets, which are likely to be adversely affecting iron (Fe) uptake and consequently chlorophyll biosynthesis [2, 3]. Besides, metal ion homeostasis and transportation would also impact the chloroplast functions, including photosynthesis [17]. Mechanistic understanding of ratoon sugarcane chlorosis may provide new perspectives to explore potential genetic solutions for this crop production constraint.

The advanced omics technologies enable a comprehensive molecular analysis of complex biological problems such as pathogenesis and nutritional disorders [18]. Transcriptome analysis provides a comprehensive gene expression profile of a particular phenotype, while metabolomics help identify changes in metabolites involved in various pathways associated with the same phenotype [19, 20]. In chlorophyll metabolism, many metabolic pathways and genes interact in a complex way [6, 19]. Comparative proteomics analysis revealed 199 and 80 proteins were down- and up-regulated in chlorotic leaves of ratoon sugarcane, respectively [16]. Through functional analysis and interaction analysis, proteins associated with photosynthesis, drought-response, and jasmonic acid biosynthesis displayed a significant correlation with ratoon sugarcane chlorosis symptoms [16]. Due to post-transcriptional modification processes, the correlation between transcriptome and proteome could be low [21]. Given this situation, exploring the mechanism underpinning ratoon sugarcane chlorosis requires both transcriptome and metabolome analyses.

Our study aimed to describe the global response of sugarcane to ratoon chlorosis through transcriptome and metabolome analyses. Genes and metabolites related to chlorophyll metabolism, photosynthesis, metal ion homeostasis, and phenylopropanoid biosynthesis were analyzed. The findings of this present a new perspective of molecular regulatory mechanisms of chlorosis occurring in ratoon sugarcane.

## Materials and methods

### Plant materials, plant growing condition, and chlorophyll content determination

A locally adapted commercial sugarcane cultivar, GuiTang49 (GT49) (ROC22 x Ganzhe 14), developed by Sugarcane Research Institute, Guangxi Academy of Agricultural Sciences, China [1, 22], was used for this study. The sugarcane experimental field was located in Fusui county, Chongzuo, Guangxi, China. All the study activities complied with the local and national legislation. For this experiment, GT49 stalks were planted on February 22, 2019, and the crop was harvested on January 31, 2021, leaving the stubbles to re-grow. When the ratoon sugarcane first displayed the chlorotic symptom, five chlorotic five non-chlorosis plants were selected randomly and grouped as chlorosis and control group, respectively. Plant height and the number of leaves and stalk nod number and tillers were recorded every 10 days for 2 months [23]. On April 18, 2021, the first expanded leaves(+ 1 leaves) of plantlets from both chlorosis and control groups were collected for further experiments.

The leaf samples from chlorosis with SPAD chlorophyll meter (Konica Minolta, Japan) reading less than ten were classified as chlorotic and used for further experiments. In contrast, leaf samples from the control group with a reading higher than 40 were grouped and used as control. Approximately 0.1 g of leaves were rinsed and ground in liquid nitrogen, and chlorophyll was extracted with 80% acetone for 24 h in dark. The content of chlorophyll was determined using a spectrophotometer [24].

### Transcriptome analysis

Five leaves were included in one sample, and each group contained five samples (5 replicates). The total RNA was extracted using Trizol (Invitrogen, USA) according to the manufacturer’s instructions. RNA quality was assessed by Agilent 2100 Bioanalyzer (Agilent, USA) and agarose gel electrophoresis. The mRNA, enriched by Oligo (dT) beads, was fragmented and reverse transcribed into cDNA first-strand with random primers. After synthesizing second-strand cDNA, the total cDNA was purified with a QiaQuick PCR extraction kit (Qiagen, The Netherlands). Sequencing adapters were ligated to the cDNA. Size selection and PCR amplification were performed before sequencing. The sequencing was operated using Illumina HiSeq 2500 (Illumina, USA).

Reads obtained from the sequencer were filtered before assembly using fastp [25]. The high-quality clean reads were processed to de novo assemble using the Trinity method [26]. The assembled reads with overlapping sequences were formed as contigs. Using clustering, the contigs that could not be extended on either end were defined as unigenes. BLASTx program, with an E-value threshold of 1e-5, was applied to annotate the obtained unigenes. Four databases, including the non-redundant protein database (NR), the Swiss-Prot database, Kyoto Encyclopedia of Genes and Genomes Ortholog (KEGG) database, and the Clusters of Eukaryotic Ortholog Groups of proteins database (KOG, NCBI), were used in the annotation of unigenes. The completeness of assembly was assessed with BUSCO (Benchmarking Universal Single-Copy Orthologs) [27]. Gene expression level was calculated using FPKM (fragments per kilobase per million reads) method with StringTie software [28]. Analyzing by DESeq software [29], genes with fold change ≥2 and *p-value* ≤ 0.05 were considered as DEGs (differential expressed genes). GO (gene ontology) and KEGG enrichment analysis was performed to depict the gene function and biological pathway of DEGs [30, 31].

### Metabolome analysis

The freeze-dry leaf samples for metabolome analysis were ground using a mixer mill (MM 400, Retsch). 100 mg of leaf powder was extracted overnight with 1.0 ml of aqueous methanol containing 0.1 mg/L lidocaine as internal standard. The supernatant was obtained by centrifugation and filtration. The metabolite compounds were analyzed by LC-ESI-MS/MS system (QTRAP 6500, Sciex, USA). The chromatographic separations were performed using a Waters ACQUITY C18 column (2.1 mm * 100 mm, 18 μm, Waters, USA) under a flow rate of 0.4 ml/min at 40 °C. The mobile phase was water (0.04% acetic acid) as Phase A and acidified acetonitrile (0.04% acetic acid) as Phase B. The separation was run in a gradient condition: 95:5 Phase A/Phase B for the first 10 min, 5:95 Phase A/Phase B for the 11th to 12th min, 95:5 Phase A/Phase B for the 13th to 15th min. The effluent was detected with Sciex Triple Quad 6500 mass spectrometer (Sciex, USA) in a positive ion mode. The quantification of metabolites was performed according to multiple reaction monitoring methods [32]. Analyst 1.6.1 software was applied for data filtering, peak detection, alignment, and calculation. Metabolites were identified by searching internal and public databases (MassBank, KNApSAcK, HMDB, MoTo DB, and METLIN) with the m/z values, retention times, and fragment patterns. Metabolites with thresholds of variable importance in projection (VIP) ≥ 1 and *p-value* ≤ 0.05 were considered to be significantly different between the chlorosis and control groups [33].

### qRT-PCR validation

The RNA used for transcriptome sequencing was also employed for qRT-PCR validation. The total RNA was digested by DNase, and reverse-transcribed into cDNA for qRT-PCR using PrimeScript RT reagent kit (TAKARA, Dalian, China). The *ACAD* gene was used as a reference gene to normalize the relative expression levels [34]. The qRT-PCR was performed on qTOWER Real-Time Thermal Cyclers (Analytik Jena, Germany). The relative expression rate was calculated with the -2ΔΔCT method [35]. A total of 20 genes, including up-and down-regulated genes, were randomly selected. All the primers of the validation gene are listed in Table S1. The correlation between transcriptome data and qRT-PCR was performed using simple linear regression analysis [36].

## Results

### Phenotype analysis of chlorotic and green ratoon plants

**Fig. 1:**
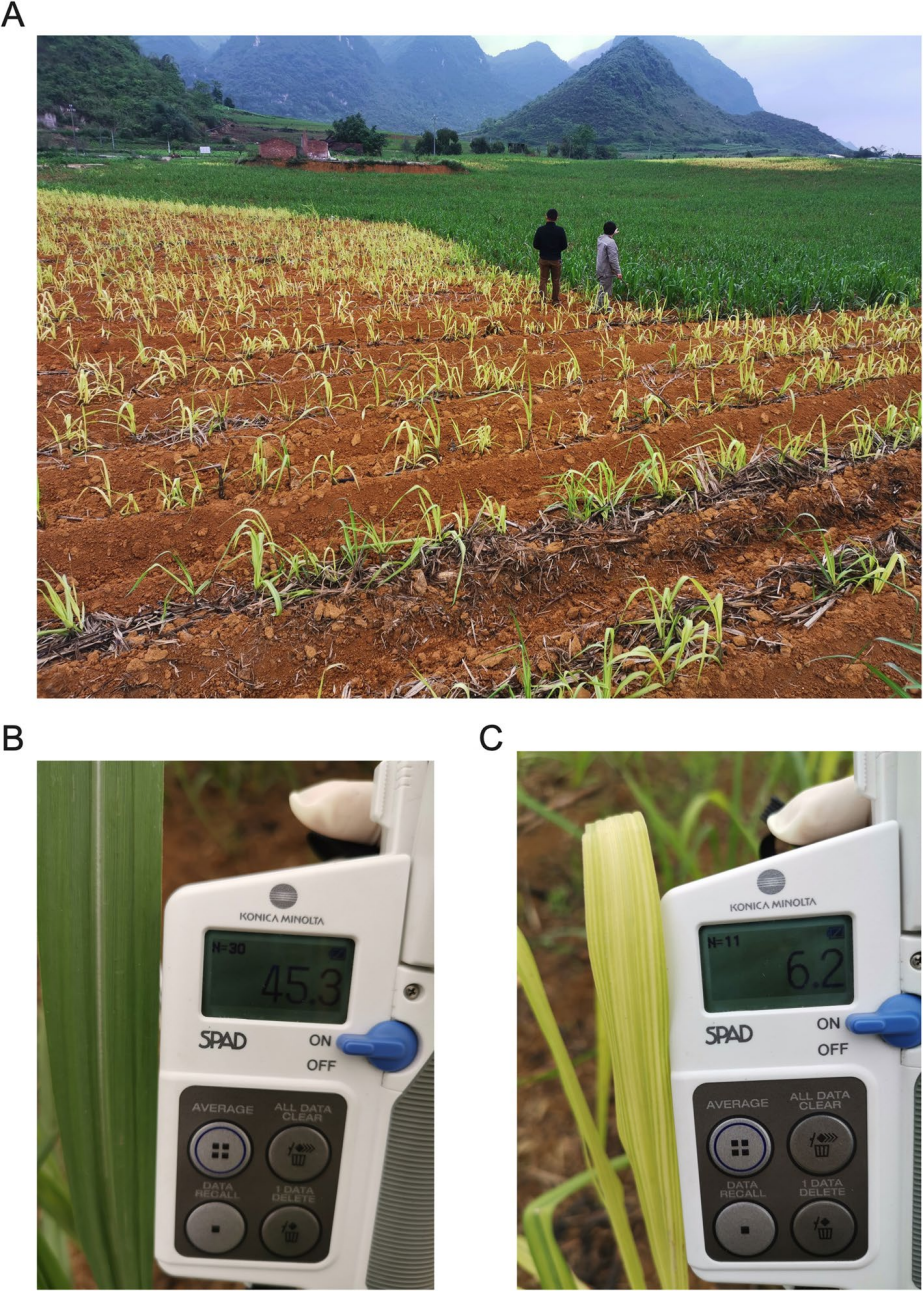
The sugarcane field with ratoon chlorotic and green plants (**A**). The green leaves (**B**, control) with SPAD reading higher than 40 and the chlorotic leaves (**C**) with SPAD reading lower than 10 were selected for experiments

**Fig. 2:**
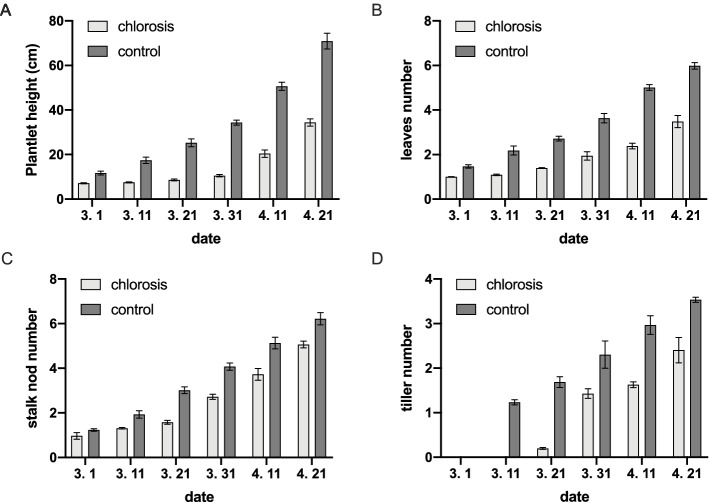
Plant height (**A**), and the number of leaves (**B**), stalk nodes (**C**), and tillers number (**D**) of plants with and without chlorosis. The x-axis represents the date of sampling

The ratoon plantlets displayed distinct chlorosis symptoms compared with the green plants found in the same field at the same time (Fig. 1A). The SPAD reading showed a drastic reduction in chlorophyll content in chlorotic leaves compared to green ones (Fig. 1B, C). To monitor the growth and development of the ratoon plants, we measured some agronomic traits. The plants showing chlorosis grew slower than the green ones. They were shorter (Fig. 2A) with fewer leaves (Fig. 2B), stalk nodes (Fig. 2C), and tillers (Fig. 2D).

### Global overview of sugarcane leaf transcriptome with chlorosis

**Fig. 3:**
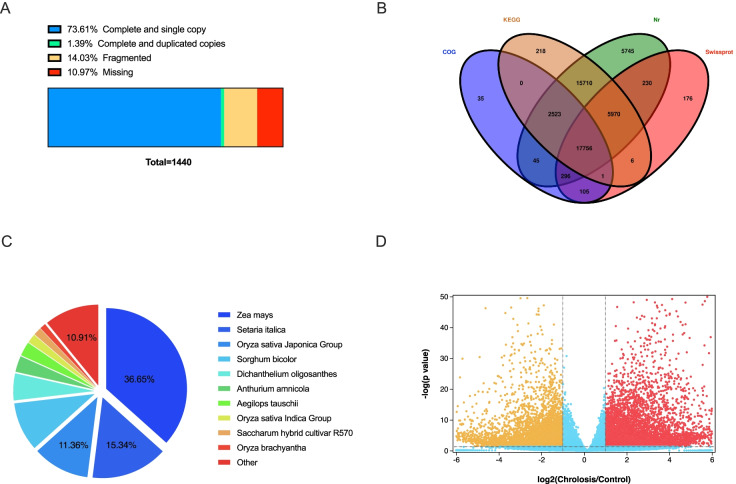
Summary of the transcriptome of sugarcane in ratoon chlorosis leaves and planted plantlet green leaves. Transcriptome de novo assembly completeness analysis using BUSCO alignment (**A**). Complete orthologues include a single copy (blue) and duplicated copies (green). Incomplete orthologues are fragmented (yellow). The missing (red) copies are not found in the BUSCO database. The Venn diagram of aligned and annotated assembly using multi databases (**B**). The distribution of species annotation unigenes (**C**). The volcano plot of expression unigenes (**D**). The up-and down-regulated genes are represented as red and yellow dots, while the light blue dots indicate the unigenes without significant changes. The unigenes with a fold change higher than 2 and a *p-value* lower than 0.05 are determined as DEGs

Since the ratoon sugarcane chlorosis is displayed conspicuously in the leaf, we hypothesized that the changes in leaf transcriptome might correlate with chlorotic responses at cellular and molecular levels. Using the same plantlets, we extracted the total RNA from the ten samples. A total of 621,045,632 reads were generated using Illumina HiSeq 4000. A total of 88,339 unigenes were de novo assembled using Trinity software. The average length of all the unigenes was 903 bp. To assess de novo assembled unigenes’ completeness, we took a quantitative measure with BUSCO (Benchmarking Universal Single-Copy Orthologs). The results showed the percentage of conserved orthologues of plants represented in our assembled sugarcane transcriptome. Completeness of all the unigenes resulted in a high rate of complete plant orthologues (75%). Of the 1440 orthologues searched in the BUSCO set of plants, 75% was complete, with the number being 1080. Of the 1080 complete BUSCO orthologues, 1060 were single-copy, while the other 20 orthologues were duplicates. The proportion of fragmented and missing BUSCOs was 14 and 11%, respectively (Fig. 3A). To annotate and classify the assembled unigenes, we employed four public protein databases to BLASTX the deduced peptides sequence of the unigenes, resulting in 48,816 unigenes being annotated (Table S2). Of all the annotated unigenes, 48,275 (98.9%) unigenes showed a particular hit within the Nr database (Non-redundant protein sequence database). Those proteins were mainly from *Zea mays*, *Setaria italica*, and *Oryza sativa* Japonica. 5745 unigenes matched the Nr database entries only, while KEGG, Swissprot, and COG had 218, 176, and 35 unigenes, respectively, and they were single matched (Fig. 3B). A total of 17,756 unigenes were found in all four databases. All the Nr hit genes were from 479 species. Of all the hits, 17,691 (36.65%) were significantly similar to proteins of *Zea mays*, and 7406 (15.34%) and 5484 (11.36%) were from *Setaria italica* and *Oryza sativa* Japonica Group, respectively (Fig. 3C). According to some criteria (log2 fold-change > 1, *p-value* ≤ 0.05), we identified a total of 11,776 differentially expressed genes (DEG) between chlorosis and control groups (Fig. 3D).

### Function analysis of DEGs in Chlorosis sugarcane

**Fig. 4:**
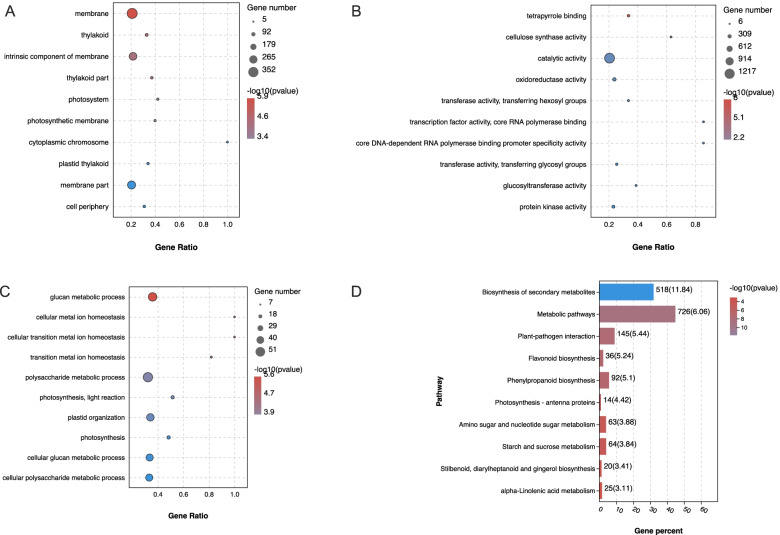
GO and KEGG pathway enrichment analysis of DEGs. The 10 most enriched GO terms in cellular component (**A**), molecular function (**B**), and biological process (**C**). The number of X axial represents the ratio of DEGs in each term. The circle size denotes gene number. The 10 most enriched KEGG pathways (**D**). The number near each column represents the gene number and percent of that pathway, respectively. High and low *p-values* are denoted in red and blue, respectively

Gene ontology (GO) analysis was employed to emphasize the function of DEGs. GO enrichment analysis revealed that DEGs were most enriched in GO cell component categories of membrane, thylakoid and intrinsic component of membrane (Fig. 4A, Table S3). In molecular function categories, tetrapyrrole binding, cellulose synthase activity and catalytic activity were the most enriched terms (Fig. 4B, Table S3). For the biological processes, glucan metabolic process (GO:0044042), cellular metal ion homeostasis (GO:0006875) and cellular transition metal ion homeostasis (GO:0046916) exhibited the highest association with DEGs of the chlorosis symptom (Fig. 4C, Table S3). To map the metabolic pathways of DEGs, we annotated these genes using KEGG analysis. A total of 31 pathways were significantly enriched from all the DEGs (Table S4). Among them, 27 pathways were related to metabolism, while two environmental information processing pathways and two organismal systems pathways were observed. Biosynthesis of secondary metabolisms, Metabolic pathways and plant-pathogen interaction were the top three enriched pathways (Fig. 4D).

### Expression of genes related to chlorophyll metabolism in chlorosis sugarcane

**Fig. 5:**
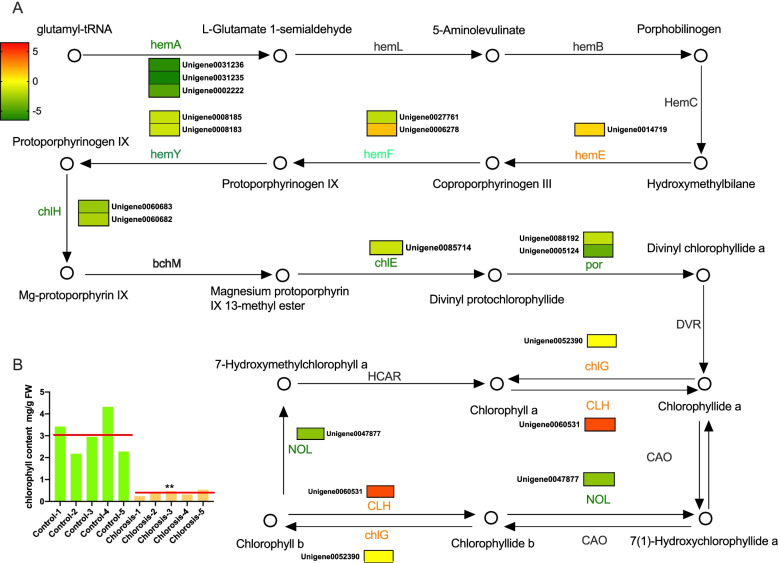
The diagram of the chlorophyll metabolism pathway. **A** The chlorophyll metabolism pathway is presented in a way of successive reaction steps. The circle denotes a chemical compound. The gene name upon the arrow denotes RNA or protein. The normalized gene expression is shown in a box with colors. The direction of the arrow means activation. Colour gradients from green to red represents the Log2FC of the genes. **B** The chlorophyll concentrations in sugarcane leaves of control and chlorosis samples. Each value represents a sample. The red line indicates the mean value of each group. An unpaired t-test was used to compare the differences between the two groups. *p-value* = 0.024. ** denotes highly significant. hemA, glutamyl-tRNA reductase; hemL, glutamate-1-semialdehyde 2,1-aminomutase; hemB, porphobilinogen synthase; hemE, uroporphyrinogen decarboxylase; hemF, coproporphyrinogen III oxidase; hemY, protoporphyrinogen/coproporphyrinogen III oxidase; chlH, magnesium chelatase subunit H; bchM, magnesium-protoporphyrin O-methyltransferase; chlE, magnesium-protoporphyrin IX monomethyl ester (oxidative) cyclase; por, protochlorophyllide reductase; DVR, divinyl chlorophyllide a 8-vinyl-reductase; chlG, chlorophyll/bacteriochlorophyll a synthase; CLH, chlorophyllase; HCAR, 7-hydroxymethyl chlorophyll a reductase; NOL, chlorophyll (ide) b reductase; CAO, chlorophyllide a oxygenase

The KEGG analysis indicated that DEGs involved in the chlorophyll metabolism pathway were enriched (*p-value* = 0.0224, Table S5). To further investigate this pathway, we found that sixteen genes were differently expressed in the chlorosis group compared to the control group, which included eleven down-regulated genes and five up-regulated genes (Fig. 5A). The down-regulated genes encode enzymes covered almost the whole process of chlorophyll biosynthesis, including glutamyl – tRNA reductase (hemA, EC: 1.2.1.70), coproporphyrinogen III oxidase (hemF, EC: 1.3.3.3), protoporphyrinogen/coproporphyrinogen III oxidase (hemY, EC: 1.3.3.4), magnesium chelatase subunit H (chlH, EC: 6.6.1.1), Mg – protoporphyrin IX monomethyl ester cyclase (chlE, EC: 1.14.13.81), protochlorophyllide reductase (por, EC: 1.3.1.33) and chlorophyll b reductase (NOL, EC: 1.1.1.294). The up-regulated genes encode uroporphyrinogen decarboxylase (hemE, EC: 4.1.1.37), chlorophyllase (EC: 3.1.1.14) and chlorophyll a synthase (chlG, EC: 2.5.1.62). Also, the content of chlorophyll in chlorosis samples (average 0.40 mg/g) was significantly lower than control samples (3.03 mg/g) (Fig. 5B). The gene expression pattern in the chlorophyll metabolism pathway, in which the majority of DEGs were down-regulated, was consistent with the reduction of chlorophyll content in chlorosis samples.

### Photosynthesis was adversely affected in chlorosis ratoon sugarcane

**Fig. 6:**
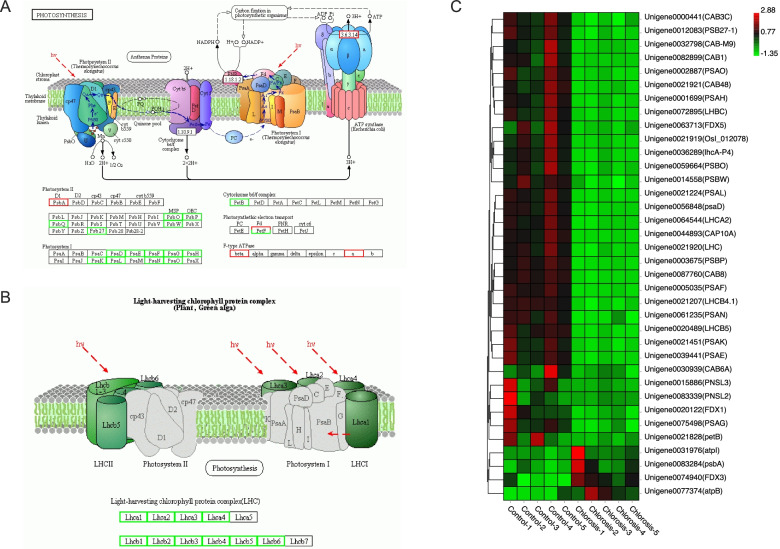
DEGs related to the photosynthesis pathways. **A** The KEGG pathway map of photosynthesis. **B** The KEGG pathway map of photosynthesis-antenna protein. The images were obtained from the KEGG database. The DEGs expression pattern was used to annotate and generate a corresponding map. The green box with gene symbols denotes down-regulated expression in the chlorosis group, while the red box denotes up-regulated expression. The genes without significant change were displayed with a grey box. **C** Expression profile of genes related to photosynthesis pathways. The vertical column represents a sample. The horizontal row represents a gene. The expression ratios are based on log2 RPKM value and normalized at row level. Each gene is presented with gene ID and gene name. PsbA-Psb27, photosystem II structure proteins; PsaA-PsaX, photosystem I structure proteins; PetB-PetG, cytochrome b6/f complex proteins; PetF, ferredoxin; beta, fF-type H+/Na + −transporting ATPase subunit beta; a, F-type H + -transporting ATPase subunit a; Lhca1-Lhca5, light-harvesting complex I chlorophyll a/b binding protein; Lhcb1-Lhcb7, light-harvesting complex II chlorophyll a/b binding protein 1

Chlorophyll is the primary pigment to capture light energy. Considering the lack of chlorophyll in the chlorosis group, we noticed that two photosynthesis pathways, including photosynthesis-antenna proteins and photosynthesis, were also enriched by KEGG analysis (Table S6). There were 36 DEGs related to KEGG photosynthesis pathways, including nine genes related to photosystem I (PSI), seven related to photosystem II (PSII), one related to cytochrome b6/f complex, three related to photosynthetic electron transport, two related to F-type ATPase, five related to light-harvesting chlorophyll protein complex I (LHCI) and nine related to LHCII (Fig. 6 A and B). Additionally, 32 genes, among the 36 DEGs of photosynthesis pathways, were significantly down-regulated in the chlorosis group, while only four genes showed up-regulated expression patterns (Fig. 6C).

### Metal ion homeostasis and transport processes under chlorosis ratoon sugarcane

**Fig. 7:**
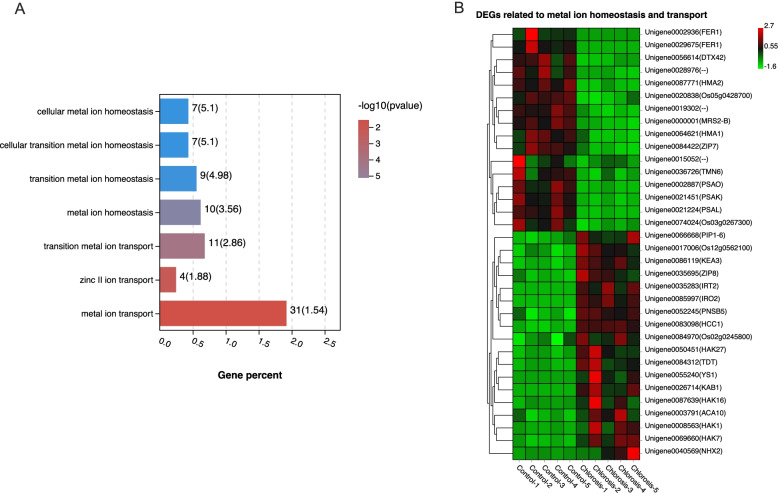
KEGG enrichment analysis and expression profiles of DEGs related to metal ion homeostasis or transport. **A** The seven enriched pathways related to metal ion homeostasis or transport (*p-value* < 0.05). The number near each column represents the gene number and percent of that pathway, respectively. High and low *p-value*s are denoted in red and blue, respectively. **B** Heat map of the expression profile of genes related to metal ion homeostasis or transport

Among the 81 enriched GO biological processes, there were four categories related to metal ion homeostasis and three categories related to metal ion transport (Fig. 7A, Table S7). In the to metal ion homeostasis category, including cellular metal ion homeostasis (GO: 0006875), cellular transition metal ion homeostasis (GO: 0046916), transition metal ion homeostasis (GO: 0055076) and metal ion homeostasis (GO: 0055065), there were 10 DEGs with six down- and four up-regulated. Of the 31 DEGs found among the three metal ion transport processes, 15 were down- and 16 were up-regulated (Fig. 7B). Homeostasis and transport processes had seven genes in common; they were two *FER1* (unigene0002936, unigene0029675), Unidentified gene (unigene0019302), *NHX2* (unigene0040569), *DTX42* (unigene0056614), *HMA1* (unigene0064621) and *HCC1* (unigene0083098). Three genes, including *TMN6* (unigene0036726), *YS1* (unigene0055240) and *IRO2* (unigene0085997), were only found in processes related to metal ion homeostasis.

### Metabolome analysis of the changes in chlorosis sugarcane

To determine the differences of metabolites in ratoon chlorosis sugarcane, we performed metabolome analysis using LC-ESI-MS/MS system. A total of 598 compounds were identified and quantified (Table S8). Thirteen compounds with VIP value ≥1 and *p-value* < 0.05 were classified as significantly differentially expressed metabolites (DEMs) between the chlorosis and control groups. All these metabolites, including six phenolic acids, three lipids, one alkaloid, one organic acid and one other compound, were down-regulated in the chlorosis group (Table 1). Among them, the compound with the greatest variation in content was coniferyl alcohol with a fold change of − 2.9. KEGG pathway analysis demonstrated that the phenylpropanoid biosynthesis pathway was the most and only enriched pathway among the different metabolites (*p-value* = 0.0088). The three compounds that belonged to the phenylpropanoid biosynthesis were coniferyl alcohol, ferulic acid and 5-O-caffeoyl shikimic acid.

**Table 1:** Differentially expressed metabolites between chlorosis and control groups

Class	Compounds	Chlorosis	Control	VIP^a^	*p-value*	Log2_Fold change
Alkaloids	p-Hydroxymandelonitrile	430,394	608,420	1.10959687	0.02027379	−0.4994096
Lipids	Octadeca-11E,13E,15Z-trienoic acid	3,844,200	4,741,460	2.56435704	0.00846175	−0.302648
	Eicosadienoic acid	461,728	577,134	1.01768916	0.00513142	−0.3218631
	LysoPC 16:0	4,335,720	6,566,380	4.19556532	0.03436214	−0.5988267
Organic acids	2-Hydroxycinnamic acid^a^	319,370	545,734	1.15424233	0.02310167	−0.7729691
Others	(S)-2-Phenyloxirane	896,878	1,115,572	1.19133863	0.02950794	−0.3148
Phenolic acids	Phthalic anhydride	710,886	857,740	1.05578543	0.02138151	−0.2709222
	Vanillin^a^	1,562,940	1,941,380	1.53827326	0.00423727	−0.3128201
	2-(Formylamino) benzoic acid	506,076	742,464	1.12174147	0.00254916	−0.552967
	Coniferyl alcohol	79,553	231,474	1.08262447	0.00357173	−1.5408619
	Ferulic acid	285,570	545,394	1.34644681	0.01281337	−0.9334544
	Dibutyl phthalate^a^	13,661,400	16,021,600	4.31621733	0.02367218	−0.2299129
	5-O-Caffeoylshikimic acid	207,131.2	406,682	1.18067851	0.01341248	−0.9733563

^a^*VIP* variable importance in projection

### The correlation of transcriptome and metabolome associated with phenylopropanoid biosynthesis

**Fig. 8:**
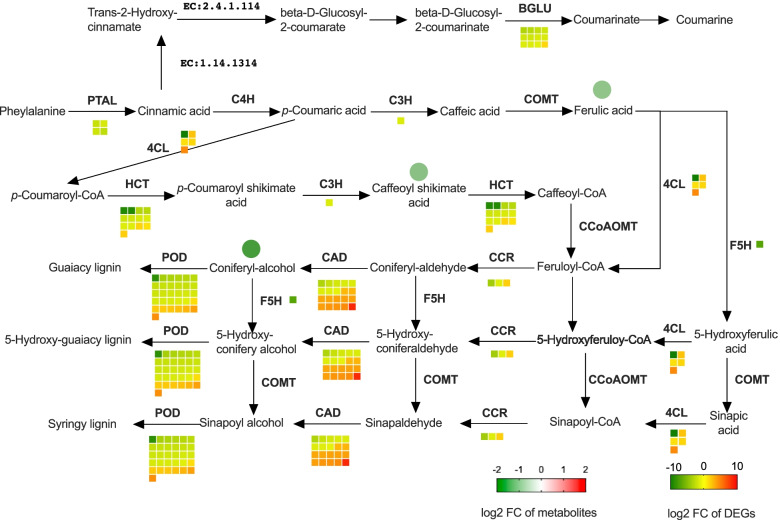
The diagram of the phenylpropanoid synthesis pathway. The names in light-type letters are metabolites compounds. The DEGs are exhibited in a bold-type letter upon the arrow. The fold change of DEGs and metabolites is shown in heatmap style. Square denotes DEGs, while the circle denotes the DEMs. Colour gradients from green to red represents the Log2FC of the genes or metabolites. PTAL, phenylalanine/tyrosine ammonia-lyase; C4H, trans-cinnamate 4-monooxygenase; C3H, 5-O-(4-coumaroyl)-D-quinate 3′-monooxygenase; COMT, caffeic acid 3-O-methyltransferase; 4CL, 4-coumarate--CoA ligase; HCT, shikimate O-hydroxycinnamoyltransferase; CCoAOMT, caffeoyl-CoA O-methyltransferase; CCR, cinnamoyl-CoA reductase; CAD, cinnamyl-alcohol dehydrogenase; POD, peroxidase; F5H, ferulate-5-hydroxylase; EC:1.14.13.14, trans-cinnamate 2-monooxygenase; EC:2.4.1.114, 2-coumarate O-beta-glucosyltransferase; BGLU, beta-glucosidase

To understand the changes of chlorosis in ratoon sugarcane, we further analyzed the correlation between metabolome and transcriptome. The only enriched pathway in differentially expressed metabolites, the phenylopropanoid biosynthesis pathway, was the fifth enriched pathway in DEGs of the transcriptome. A total of 92 genes were involved with the phenylpropanoid biosynthesis pathway among the 11,776 DEGs, while three out of 13 differential metabolites belonged to this pathway. Sixty-two genes out of the 92 DEGs, were down-regulated (Fig. 8, Table S9). In our transcriptome, the *POD* gene, encoded a peroxidase, was identified expressed by 31 DEGs with 23 down-regulated and 8 up-regulated. Also, DEGs encoded *HCT*, *PTAL*, *C3H*, *CCR*, *F5H*, *BGLU* were mainly down-regulated in the chlorosis group. In contrast, we observed more up-regulation in *CAD* and *4CL*. The down-regulation of the two essential genes, *PTAL* and *C3H*, could largely explain the low accumulation of ferulic acid and 5-O-caffeoyl shikimic acid (Table 1). Furthermore, the low accumulation of coniferyl-alcohol and down-regulation of *POD* and *F5H* indicated that the lignin biosynthesis was also impaired.

### qRT-PCR analysis

**Fig. 9:**
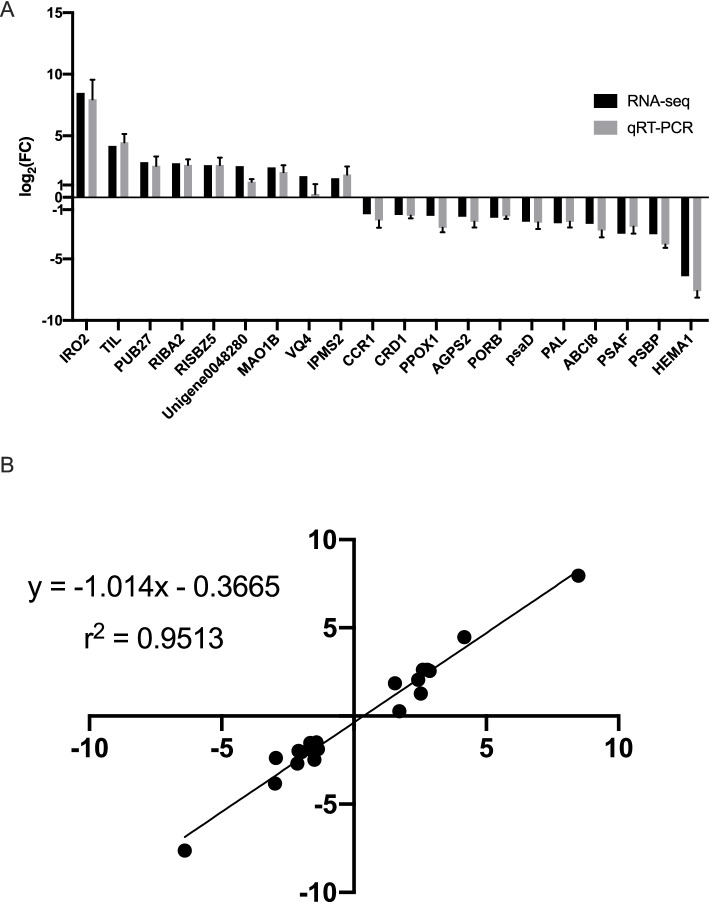
The validation of transcriptome using qRT-PCR. **A** The 20 genes expression pattern of transcriptome and qRT-PCR. The columns in black and grey denote the expression value of transcriptome and qRT-PCR, respectively. The value represents the log2 fold change in the chlorosis group compared with the control group. **B** Correlation of transcriptome (x-axis) and qRT-PCR (y-axis) data

To validate the DEGs expression pattern, we randomly selected 20 genes, including up-and down-regulated ones with high and low expression rates, for quantitative real-time (qRT)-PCR assays. The primary expression trend of the 20 genes was consistent (Fig. 9A). The value of transcriptome and qRT-PCR showed a significant positive correlation with the Pearson r-value of 0.9867. The linear regression of correlation analysis was conducted, resulting in the goodness of fit with an R square value of 0.9513 (Fig. 9B). These results indicated that the transcriptome was reliable.

## Discussion

Ratooning is a cultivation method in sugarcane production [37]. Ratoon sugarcane chlorosis, despite reporting several decades ago, is still a severe problem, causing a significant economic loss in China [3, 16]. The main reason for ratoon sugarcane chlorosis is considered to be the imbalance between Fe and Mn concentration, which may be induced by acidic soil, intense leaching, and continuous cultivation [2, 3]. Although causes and treatments have been studied previously, the associated regulatory mechanisms of ratoon sugarcane chlorosis remain unclear. In this study, RNA-Seq was employed to demonstrate the global transcriptomic changes of ratoon sugarcane with chlorosis. DEGs related to chlorophyll metabolism, photosynthesis, and metal ion homeostasis were significantly affected by chlorosis. In addition, combining metabolome analysis, DEGs and DEMs were enriched in the phenylopropanoid biosynthesis pathway.

Chlorophyll is the primary source of pigment in plant leaves, which is correlated with the green color [38]. The well-studied chlorophyll synthesis pathway involved many essential genes [4, 39]. HemA, which catalyzes the glutamy-tRNA to L-Glutamate 1-semialdehyde, is the initial enzyme of chlorophyll synthesis that regulates the chlorophyll accumulation during de-etiolation [40]. HemY catalyzes the protoporphyrinogen-IX to protoporphyrin-IX, which is predominant in chlorophyll synthesis [41]. The magnesium chelatases subunit H, named chlH, catalyzes the magnesium ion and protoporphyrin-IX to form Mg-protoporphyrin-IX, which was found induced by light [42]. Knock-down of chlE, an Mg-protoporphyrin-IX monomehyl ester cyclase, resulted in retarded growth and chloroplast developmental defects in Arabidopsis [43]. The protochlorophyllide reductase (por), the enzyme that promotes a photoreduction of protochlorophyllide to chlorophyllide, plays a vital role in the greening stage [44]. NOL, a chlorophyll b reductase, plays a role in chlorophyll b degradation [45]. In this study, twelve down-regulated DEGs, including three hemA, one hemF, two hemY, two chlH, one chlE, two por, and one NOL, were found associated with the chlorophyll synthesis pathway (Fig. 5). Compared to the four up-regulated DEGs, including one hemF, one hemE, one chlG, and one CLH, the down-regulated genes in this pathway were in the majority. The same phenomenon was observed in wheat yellow leaves and light green cucumber [6, 46]. These results indicate the direct relation between ratoon sugarcane chlorosis and the dysfunction of chlorophyll synthesis.

Leaf photosynthesis is positively correlated with chlorophyll content [47]. In *A. thaliana*, photosynthetic efficiency reduction was accompanied by a decrease in chlorophyll content [48]. In wheat yellow leaves wheat, five genes encoding photosynthesis related proteins were significantly down-regulated [46]. Transcriptome and proteome analysis of a wheat mutant with albino leaves showed that the expression levels of both genes and proteins related to photosynthesis were lower than wild-type green wheat [49]. Likewise, we observed similar results in this study. There were 36 DEGs found in photosynthesis pathway, of which 32 were down-regulated (Fig. 6). The light-harvesting chlorophyll protein complexes (LHC) binds chlorophyll to function in the photosynthesis system [50]. Lack of the LHC would affect plants’ photosynthetic rate and growth [51]. In chlorosis sugarcane, we found that all the DEGs of LHC were down-regulated (Fig. 6), suggesting that the formation of photosynthesis’s antenna proteins would be affected. Photosynthesis occurs in the chloroplast, which comprises chloroplast membrane, thylakoid, and matrix. The multi subunits complexes, including PSI, PSII, cytochrome b6/f complex, and photosynthetic electron transport, are embedded in the thylakoid membrane [52]. DEGs of these complexes were significantly repressed in chlorosis sugarcane (Fig. 6). The GO enrichment analysis of cellular components showed that five GO terms, including thylakoid, thylakoid part, photosystem, photosynthetic membrane, and plastid thylakoid, were among the top 10 enriched (Fig. 4A). These results agreed with previous reports in wheat where yellow leaf mutant was caused by abnormal chloroplast development [53].

Studies of ratoon sugarcane chlorosis in Guangxi suggest that the excessive Mn accumulation in parent stalks was the main reason for chlorosis [3]. At the same time, the active Fe deficiency in plantlets played a secondary role [3]. The subsequent investigation revealed that, in the progression of the greening of chlorotic seedling, the leaf Mn content decreased significantly, whereas Fe content increased [54]. Mn shares similar chemical properties with Fe and Mn, and Fe interaction was observed in several physiological processes [55]. Mn transport is partially mediated by Fe transporter [56], and thus Mn accumulation in ratoon sugarcane may play a role in Fe deficiency. These findings are consistent with our results on metal ion metabolism disorder related gene expression in chlorosis sugarcane. Our GO analysis has shown that seven categories related to metal ion homeostasis and transport were significantly enriched (Fig. 7A). A total of 34 DEGs were found in these categories (Fig. 7B). Iron-phytosiderophore transporter (YS1) and transcription factor bHLH100 (IRO2) were up-regulated by iron deficiency [57, 58]. Ferritin 1 (FER1), important for iron homeostasis, stores iron in a soluble form, is up-regulated by iron overload treatment [59]. In our study, two genes, YS1(unigene0055240) and IRO2(unigene0085997), were significantly increased with log2 FC higher than 8, while FER1 (unigene0002936, unigene0029675) were down-regulated with log2 FC lower than − 6 (Table S7). Also, MRS2-B (Unigene0000001), a magnesium transporter, was found down-regulated in the chlorosis group (Fig. 7B, Table S7). These results indicated that the differential regulation of metal ion metabolism genes agreed with the imbalance of Fe and Mn. However, whether the asymmetry of metal ion induces differential gene expression or do the differentially expressed genes lead to iron deficiency in the leaf remains unclear.

In a previous study, the phenylpropanoid biosynthesis pathway was enriched in both transcriptome and metabolome analysis of chlorosis *A. catechu* L. [5]. Combining transcriptome and metabolome analyses, we found 62 DEGs and 3 DEMs (differentially expressed metabolites) belong to the phenylpropanoid biosynthesis pathway (Fig. 8, Table S9, and Table 1). Interestingly, phenylpropanoid biosynthesis was the only enriched pathway in DEMs. These results indicate that phenylpropanoid biosynthesis could be involved in ratoon sugarcane chlorosis development. Phenylpropanoids contribute significantly to plants’ response towards biotic and abiotic stresses [60]. In chlorotic tea leaves, genes and metabolites related to phenylpropanoids biosynthesis showed lower expression than green leaf [61]. In an iron deficiency chlorosis tolerant soybean, genes in the phenylpropanoid biosynthesis were up-regulated in low Fe condition [62]. Synthesis of coumarins is part of Fe acquisition machinery in *Arabidopsis* [63]. We noticed the *BGLU*, a beta-glucosidase coding gene, was down-regulated in chlorosis sugarcane (Fig. 8). This result suggests that the phenylpropanoid biosynthesis was altered at the transcripts and metabolites level in chlorotic ratoon sugarcane. Given the central role of phenylpropanoid biosynthesis in plant growth and development [60, 64], two secondary metabolic pathways, including flavonoids and phenylalanine, were found enriched in KEGG analysis (Fig. 4D, Table S4).

## Conclusions

Here we studied transcriptome and metabolome responses of chlorotic and non-chlorotic sugarcane leaves. Our results provide more insights into chlorophyll synthesis, photosynthesis, metal ion metabolism, and phenylpropanoids biosynthesis in ratoon chlorosis sugarcane. Gene regulation dysfunction in metal ion homeostasis and transportation likely leads to Mn toxicity and Fe deficiency, which may cause ratoon chlorosis in sugarcane [3]. The reduction of chlorophyll content is likely due to the decreased chlorophyll synthesis pathway. Furthermore, the photosynthesis related genes were also affected significantly. The consistent results of metabolome and transcriptome of phenylpropanoids biosynthesis pathway suggest that the alteration in secondary metabolism plays a vital role in ratoon sugarcane chlorosis.

## Supplementary Information

**Additional file 1: Table S1.** The primers for validation of transcriptome. The table included the twenty genes that selected for validation of transcriptome. The parameter of each primer, which included primer sequence, melting temperature, product length and so, were also listed. The FPKM value, GO and KEGG annotation of each gene was indicated.

**Additional file 2: Table S2.** The expression and annotation of all the differential expression unigenes. This table included 11,776 unigenes. The read count, FPKM value, foldchange, KEGG and GO annotation were indicated.

**Additional file 3: Table S3.** The GO enrichment analysis results of the differential expression unigenes.

**Additional file 4: Table S4.** The KEGG pathway enrichment analysis results of the differential expression unigenes.

**Additional file 5: Table S5.** The differential expression genes of Porphyrin and chlorophyll metabolism pathway.

**Additional file 6: Table S6.** The differential expression genes of Photosynthesis and Photosynthesis - antenna proteins pathways.

**Additional file 7: Table S7.** The differential expression genes related to metal ion homeostasis and transport process.

**Additional file 8: Table S8.** The 598 metabolites identified in metabolome. The table included the quantification value, mass spectrum character, and compound identification information of each metabolites.

**Additional file 9: Table S9.** The differential expression genes of Phenylpropanoid biosynthesis pathways.

## Data Availability

All data generated in this study are included in this published article and the relevant additional files. The transcriptome raw reads generated in this study have been deposited in BioProject with the accession number of PRJNA787323 (https://www.ncbi.nlm.nih.gov/bioproject/PRJNA787323). Requests for material should be made to the corresponding author.
